# The Roles of Tenascins in Cardiovascular, Inflammatory, and Heritable Connective Tissue Diseases

**DOI:** 10.3389/fimmu.2020.609752

**Published:** 2020-12-01

**Authors:** Ken-ichi Matsumoto, Hiroki Aoki

**Affiliations:** ^1^ Department of Biosignaling and Radioisotope Experiment, Interdisciplinary Center for Science Research, Organization for Research and Academic Information, Shimane University, Izumo, Japan; ^2^ Cardiovascular Research Institute, Kurume University, Kurume, Japan

**Keywords:** tenascin-C, tenascin-X, cardiovascular disease, fibrosis, inflammation, Ehlers-Danlos syndrome

## Abstract

Tenascins are a family of multifunctional extracellular matrix (ECM) glycoproteins with time- and tissue specific expression patterns during development, tissue homeostasis, and diseases. There are four family members (tenascin-C, -R, -X, -W) in vertebrates. Among them, tenascin-X (TNX) and tenascin-C (TNC) play important roles in human pathologies. TNX is expressed widely in loose connective tissues. TNX contributes to the stability and maintenance of the collagen network, and its absence causes classical-like Ehlers-Danlos syndrome (clEDS), a heritable connective tissue disorder. In contrast, TNC is specifically and transiently expressed upon pathological conditions such as inflammation, fibrosis, and cancer. There is growing evidence that TNC is involved in inflammatory processes with proinflammatory or anti-inflammatory activity in a context-dependent manner. In this review, we summarize the roles of these two tenascins, TNX and TNC, in cardiovascular and inflammatory diseases and in clEDS, and we discuss the functional consequences of the expression of these tenascins for tissue homeostasis.

## Introduction

An important component of the extracellular environment is the extracellular matrix (ECM), which is comprised of glycoproteins, proteoglycans, and fibrillar proteins. The ECM offers not only structural support for cells but also influences cell adhesion, proliferation, differentiation, and survival through specific receptor-mediated interactions (1). Within the ECM, the tenascins comprise an attractive glycoprotein family with distinct features for each member.

Tenascins comprise four members in vertebrates: tenascin-C (TNC), tenascin-R (TNR), tenascin-X (TNX) [referred to as tenascin-Y (TNY) in chickens], and tenascin-W (TNW) (originally named tenascin-N in mice) (2, 3). The tenascin family members have a common structure with heptad repeats, epidermal growth factor (EGF)-like repeats, fibronectin type III (FNIII)-like repeats, and a fibrinogen (FBG)-related domain. This modular structure allows tenascins to interact with multiple binding partners, including cell surface receptors, cytokines, and extracellular matrix molecules. Each of tenascins shows a unique time- and tissue specific expression pattern both during development and in adulthood (4–8). On the other hand, tenascins are also subjected to dynamic remodeling during a number of pathological conditions such as inflammation, fibrotic disorders, cardiovascular diseases, and cancer progression (9). Transcriptional control of tenascin family members for their specific expression patterns has recently been reviewed (10). Such an expression pattern of tenascins is one of the features of all matricellular proteins including tenascins (11, 12).

## TNX

### Expression of TNX in Physiological and Pathological Conditions

#### Regulation of TNX Expression

TNX expression is undetectable during early embryonic stages, but its expression increases ubiquitously in various tissues, especially in heart, skeletal muscle, and skin, during the middle embryonic stage and after birth (13–15). TNX is associated with blood vessels in most tissues and its distribution is often reciprocal to that of TNC, particularly in the skin and tissues of the digestive tract (13). Interestingly, by the analyses of TNC-deficient mice it was found that TNX does not compensate for the loss of TNC, at least in the brain (16) and during early heart development (17).

As for the regulation of TNX expression by the cellular microenvironment, brain-derived neurotrophic factor (BDNF) stimulates its mRNA expression in endothelial cells (18), whereas TNX is subjected to downregulation by glucocorticoids in fibroblasts (19). Sp1, which is a widely distributed transcription factor, is essential for expression of the mouse TNX gene (*Tnxb*) (20). Recently, microRNA miR-30b (21), long non-coding RNA (lncRNA) LINC01305 (22, 23), and circular RNA (circRNA) circRNA_14940 (24) have also been revealed to be key regulators of TNX expression.

#### TNX Expression in the Nervous System

Recently, the expression pattern and significance of TNX in the nervous system have become apparent. In the nervous system, TNX is localized in the perineurium and endoneurium of the peripheral nervous system (PNS) such as sciatic nerves (15, 25). Indeed, patients with TNX-deficient type EDS (classical-like EDS: clEDS) show abnormal peripheral nerves (26). TNX has been expressed in Schwann cells but not in axons (27). TNX has been mainly detected in the leptomeninges in the spinal cord and in the pia matter of the dorsal root ganglion (DRG). In the DRG, TNX is localized in satellite cells surrounding primary sensory neurons (27). In the central nervous system (CNS), TNX has been detected in the leptomeninges and choroid plexus of the adult cerebral cortex (28). Avian TNX (TNY) has been shown to inhibit neurite outgrowth and reduce the spread of growth cones (29).

#### TNX Expression in Cancers

Although there have been fewer reports on TNX expression in cancer compared with reports on the expression of TNC and TNW in cancer, reports on TNX expression have been increasing. TNX has been shown to be highly expressed in malignant mesothelioma (30, 31) and ovarian cancer (32), indicating the possibility of TNX being a novel diagnostic maker of these cancers. On the other hand, there have been several reports of TNX expression being downregulated during tumor progression in astrocytomas (33), cutaneous melanoma (34), and neurofibromatosis type 1 (35), findings that are mostly opposite to those for TNC. Intriguingly, it has also been reported that TNX has a tumor suppressor role in cervical cancer *via* LINC01305 expression which modulates TNX expression (22), esophageal squamous-cell carcinoma (36), and lung cancer *via* LINC01305 expression (23) and that TNX is downregulated in these tumors. In agreement with the tumor suppressor role of TNX in cancer progression, TNX-deficient mice with grafted melanoma cells exhibited promotion of tumor invasion and metastasis because of increased activities of matrix metalloproteinases (MMPs) (37, 38). Interestingly, by the analyses of TNX and TNC single and/or double deficient mice, we found out that TNX deficiency-induced tumor cell proliferation in the primary tumor site is repressed by the lack of TNC, while TNX deficiency-induced invasion to neighboring tissues is not promoted by the lack of TNC (39).

### Physiological Functions of TNX

The results of a number of studies on abnormalities in mice with targeted deletion in *Tnxb* (40) and in clEDS patients (26, 41) have suggested structural roles of TNX in tissue integrity (7, 42). TNX possesses elastic properties in the FNIII-like domain (43) and increases the stiffness of collagen gels (44). TNX is associated with collagen fibrils within tissues and regulates collagen fibril spacing (42) *via* direct interaction with types I, III and V fibrillar collagens (45), types XII (46) and XIV fibril-associated collagens (45), and decorin (47). It has also been shown that TNX increases both the rate and extent of fibril formation *in vivo*, indicating a crucial role of TNX in collagen fibrillogenesis (48, 49). Taken together, the findings suggest that TNX regulates collagen deposition, collagen fiber stability and collagen mechanical properties. In addition, it has been shown that TNX binds to tropoelastin (49). Coarse and fragmented immature elastin fibers have been detected in clEDS patients, suggesting that TNX is also involved in the stability and maintenance of elastin fibers (50).

### Other Functions of TNX

Fragments of TNX, especially its EGF-like repeats and FNIII-like repeats, have profound proangiogenic properties (51). Furthermore, we have shown that TNX interacts with vascular endothelial growth factor B (VEGF-B) and stimulates endothelial cell proliferation *via* simultaneous binding to VEGF receptor 1 (VEGFR-1) and VEGF-B (52). Indeed, results of *in vivo* studies using TNX-deficient mice have shown that TNX plays a crucial role in blood vessel formation in sciatic nerves (53) and in injury-induced stromal angiogenesis in the cornea (54). Recently, we have reported that TNX-deficient mice display upregulation of osteoclast marker gene expression and promoted bone resorption activities due to increased multinucleated osteoclasts (55). These results provide the first evidence for the essential functions of TNX in bone metabolism such as osteoclast differentiation. These non-structural functions of TNX may be related to the structural roles of this ECM glycoprotein. The modification of the composition and organization of extracellular environment due to TNX deficiency might cause the alteration of mechanical stress to the surrounding cells, leading to the non-structural aberrations.

Alcaraz *et al (* 56 *).* demonstrated that the C-terminal FBG-related domain of TNX activates the latent transforming growth factor-β (TGF-β) into the active molecule and that integrin α11β1 is required as a cell surface receptor for TNX for this activation. They also showed that the FBG-related domain-mediated TGF-β activation elicits the TGF-β/Smad signaling pathway and causes epithelial-mesenchymal transition (EMT) in epithelial cells (56).

So far, a number of important phenotypes have been observed by studying TNX-deficient mice (**Table 1**).

**Table 1:** Tenascin-X-deficient mouse phenotypes.

Phenotypes		References
clEDS-related phenotypes	Hyperextensible skin, reduced tensile strength, reduced collagen deposition and stability, reduced fibrillar collagen, increased elastic fibers	(40, 48, 49, 57),
	Muscle weakness, myopathic changes	(58, 59),
	Reduced diameter of myelinated fibers in sciatic nerves	(59)
	Abnormal wound healing	(60, 61),
	Gastrointestinal pain and dysfunction, increased colonic afferent sensitivity and increased sensory neuronal sprouting	(62, 63),
	Mechanical allodynia and hypersensitivity to chemical stimuli	(27)
	Abnormal location of vaginal plug, rectal prolapse	(64)
Behavior	Increased anxiety, superior memory retention, increased sensorimotor coordination	(65)
Blood vessel formation and neovascularization	Abnormal blood vessel formation and less neovascularization	(53, 54),
Triglyceride synthesis	Accumulation of triglycerides and altered composition of triglyceride-associated fatty acids	(66)
Bone homeostasis	Bone loss due to increased osteoclastogenesis	(55)
Tumor progression	Promotion of invasion and metastasis of melanoma cells, increased activities of MMPs	(37, 38),
Liver fibrosis	Suppression of hepatic dysfunction by administration of a high-fat diet	(67)

### clEDS Caused by TNX Deficiency

Ehlers-Danlos syndrome (EDS) is a group of clinically and genetically heritable connective tissue disorders characterized by joint hypermobility, skin hyperextensibility, and generalized connective tissue fragility (68). So far, EDS has been classified into 14 distinct subtypes caused by defects in 20 different genes encoding fibrillar collagens and collagen-modifying proteins and ECM proteins (69, 70). Among the subtypes, classical-like EDS (clEDS) is caused by a complete lack of TNX due to homozygous or compound heterozygous TNX gene (*TNXB*) mutations with autosomal recessive inheritance, leading to nonsense-mediated decay of the mutant RNA (41). clEDS shows typical clinical hallmarks characterized by soft/velvety hyperextensible skin without atrophic scarring, generalized joint hypermobility and easy bruising as its major clinical features (41). TNX is also present in sera. The serum form of TNX (sTNX) with a molecular size of 140 kDa is generated by cleavage of the 450-kDa mature form of TNX (41). The measurement of sTNX concentration is useful for the diagnosis of clEDS (71).

Mitral valve abnormality (24%) and hypertension (24%) have been reported as cardiovascular complications in clEDS patients (72). It has also been reported that clEDS patients exhibit rectal prolapse (18%) and diverticulosis or diverticulitis (18%) as gastrointestinal complications (72). Currently, these gastrointestinal complications are considered to be more common in clEDS patients (73, 74).

Nearly 90% of patients with EDS show chronic pain (75). clEDS patients frequently complain of chronic back pain, chronic myalgia and chronic arthralgia (72). Recent investigations of TNX-deficient mice have shown that there is a direct link between TNX deficiency and pain. For example, Aktar *et al.* showed that TNX-deficient mice have hypersensitive colonic nociceptive afferents and increased sensory neuronal sprouting, leading to gastrointestinal pain and dysfunction (62). In addition, we recently reported that TNX-deficient mice exhibit mechanical allodynia and hypersensitivity to chemical stimuli and hypersensitization of myelinated Aδ and Aβ fibers (27).

### TNX and Fibrosis

In a previous study, we showed that TNX contributes to liver fibrosis in TNX-deficient mice administered a high-fat and high-cholesterol diet with high levels of phosphorus and calcium (HFCD) (67). Inflammation assessed by inflammatory cell infiltrates and levels of type I collagen was suppressed in TNX-deficient mice compared with that in wild-type mice. On the other hand, the TGF-β pathway is a well-known key signaling pathway associated with hepatic stellate cell activation and fibrosis progression (76). As mentioned above, TNX affects latent TGF-β activation and signaling (56). Thus, it is reasonable to assume that TNX, especially its FBG-related domain, contributes to liver fibrosis and inflammatory responses *via* the TGF-β pathway in combination with integrin α11β1.

### Other Diseases Associated With Mutations or SNPs in *TNXB*

It has been reported that another disease associated with heterozygous mutations in *TNXB* is primary vesicoureteral reflux (VUR) (77). There is also some evidence that single nucleotide polymorphisms (SNPs) in *TNXB* are associated with other diseases. For example, genomic studies with SNPs in genome-wide association studies revealed that two closely linked SNPs in the coding region of *TNXB* are associated with schizophrenia risk in a Japanese population by a case-control study (78). On the other hand, an SNP in the 5’ flanking region of *TNXB* has been reported to be associated with systemic lupus erythematosus (SLE) (79). However, the functional implications of SNPs in *TNXB* relevant to these diseases remain uncertain and warrant further investigation.

## TNC

### Context-Dependent Function of TNC

TNC is a prototypical and most well-characterized member of the tenascin family. TNC has a variety of biological functions including regulation of cell adhesion, migration, growth and differentiation by binding through its modular structure to multiple cell surface receptors including integrins, Toll-like receptor 4 (TLR4) and syndecan-4 (80, 81). TNC also binds to cytokines such as fibroblast growth factors (FGFs), platelet-derived growth factors (PDGFs) and TGF-β family members among others, thus regulating the cellular behavior and organization of the extracellular matrix.

The expression of TNC is regulated during embryonic development with a specific time and spatial pattern, and its expression is greatly diminished in adult tissue. Although the specific expression pattern of TNC was suggestive of its role in embryogenesis, mice with genetic deletion of TNC were born and grew without any gross abnormality and were fertile (16). Later, it was demonstrated that TNC-deficient mice exhibit abnormalities in their behavior and in the cytoarchitecture of the brain (82). Considering the extensive expression of TNC during embryogenesis, TNC may have more roles in fine tuning animal development that are yet to be clarified.

TNC is transiently and specifically re-expressed upon acute inflammation and is persistently expressed upon chronic inflammation (83–85). Growing evidence has suggested that TNC is a proinflammatory factor and plays a deleterious role in fibrotic diseases (86–88). Interestingly, several lines of evidence have suggested that TNC also acts as an anti-inflammatory factor. For example, it was shown that the first two alternative spliced FNIII-like repeats suppress *in vitro* T cell activation (89). The results of *in vivo* studies showed that chemically induced inflammatory dermatitis (90) and Habu-snake venom-induced glomerulonephritis (91) develop more severely in TNC-deficient mice than in wild-type mice. Such bimodal activities, namely proinflammatory or anti-inflammatory activities, of TNC can have paradoxical effects and may influence many aspects of the immune response in a context-dependent manner.

The context-dependent function of TNC seems to be derived from its multidomain structure, which allows TNC to interact with multiple extracellular matrix and cytokines (81). In addition, TNC gene can generate multiple variants of TNC protein by alternative splicing of mRNA in tissue- and disease-specific manners (92), and proteolytic processing by various proteases, of which significance has been demonstrated by experiments with domain-specific antibodies and recombinant proteins.

### Pathophysiological Role of TNC in Cardiac Diseases

TNC is reported to be involved in a variety of cardiovascular diseases (83, 93, 94). In the pathogenesis of myocardial damage and cardiac dysfunction, animal experiments have demonstrated that TNC is involved in adverse remodeling of myocardium due to myocardial infarction (95, 96) and myocarditis (97). TNC has been reported to promote myocardial hypertrophy, fibrosis (98, 99) and cardiac dysfunction (100) in animal models of cardiac hypertrophy and myocardial infarction. Being consistent with those findings, TNC has been shown to promote cardiac fibrosis in an angiotensin II-induced hypertrophy model (101). However, another study showed that TNC attenuated cardiac fibrosis due to pressure overload or angiotensin II infusion (102). These contradictory findings may be due to the fact that the adverse effect of TNC was demonstrated in a BALB/c background of the mouse strain, while its beneficial effect was shown in the background of C57BL/6. It was further speculated that the difference may reflect the predominant immune responses of Th2 in BALB/c and Th1 in C57BL/6, although this hypothesis awaits formal proof (103).

### Pathophysiological Role of TNC in Vascular Diseases

**Figure 1:**
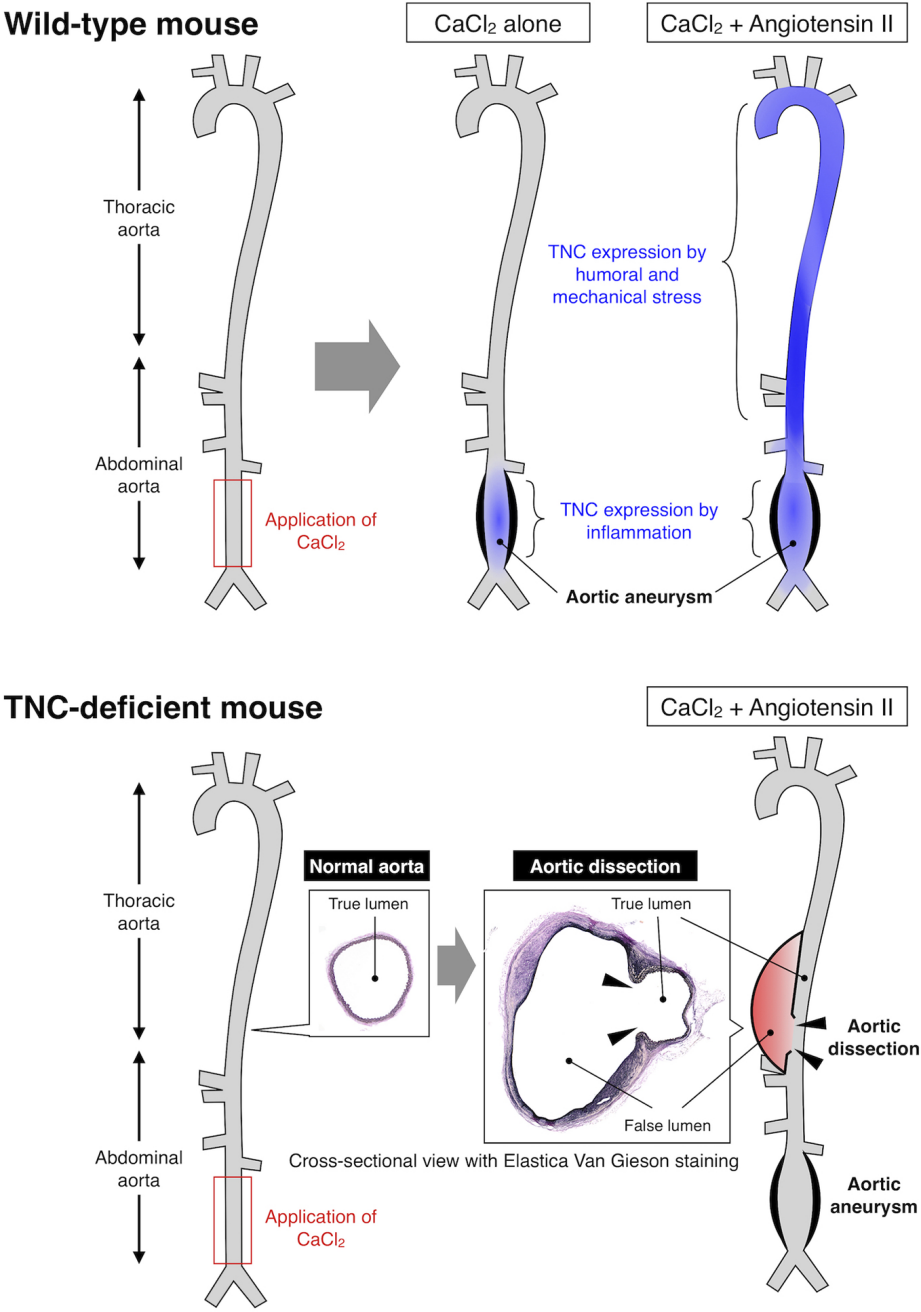
Expression and function of TNC in mouse model of aortic diseases. Upper panel: Application of CaCl_2_ solution to the lower abdominal aorta caused local inflammation and formation of aortic aneurysm. Continuous infusion of angiotensin II after the CaCl_2_ application resulted in higher wall stress and formation of larger aortic aneurysm. TNC was induced in the lower abdominal aorta by CaCl_2_-induced local inflammation, and in the thoracic and upper abdominal aorta due to the angiotensin II-induced higher wall stress, as illustrated by the blue color. Lower panel: TNC-deficient mice developed aortic aneurysm comparable to wild-type mice by CaCl_2_ application in the presence or absence of angiotensin II. On the other hand, TNC-deficient mice developed aortic dissection in the thoracic and upper abdominal aorta that was characterized by the disruption of the aortic wall (arrowheads) and the formation of false lumen (red color). These findings indicate that TNC does not play a major role in the destructive inflammation in the aortic aneurysm, while it is critical for protecting the aortic wall from dissection, exemplifying the context-dependent function of TNC (111).

With regard to vascular diseases, TNC has been reported to be atherogenic by stimulating TLR4-dependent foam cell formation (104). However, TNC has also been reported to be anti-atherogenic since TNC-deficient mice showed mast cell accumulation and intraplaque hemorrhage (105, 106). Similarly, expression of TNC may prevent the rupture of cerebral aneurysm by promoting fibrosis of the aneurysmal wall (107, 108), while it may be deleterious by exacerbating acute vasospastic response and exacerbate cerebral injury after subarachnoidal hemorrhage (109, 110). Expression of TNC by neurohumoral stress protects the aorta from acute aortic dissection (111) (**Figure 1**), while it seems to have no impact on the development of abdominal aortic aneurysm, although it was highly expressed in the aneurysmal tissue (112). Therefore, TNC can be either disease-promotive, disease-preventive or neutral in cardiovascular diseases (94), underscoring the context-dependent function of TNC, as demonstrated also in various animal models of non-cardiovascular diseases.

### TNC as a Biomarker of Tissue Damage

While the role of TNC is context-dependent and can be detrimental or beneficial, it has been established that TNC is expressed in various cardiovascular diseases in clinical settings (93). TNC is elevated after myocardial injury due to myocardial infarction (113) or due to acute (114) or chronic myocarditis (115). TNC is also elevated in hypertrophic (116) and dilated cardiomyopathies (117). In addition, TNC is elevated in heart failure with preserved ejection fraction (HFpEF) (118) and in right ventricular failure (119). TNC is not only deposited in the damaged tissue but also liberated in circulating blood flow. It has been proposed that TNC can serve as a prognostic marker for heart failure due to these diseases. While B-type natriuretic peptide (BNP) is an established prognostic marker for heart failure, the combination of BNP and TNC may be more precise than BNP or TNC alone for patients with dilated cardiomyopathy (120). Furthermore, reverse remodeling of the ventricle in heart failure patients due to cardiac resynchronization therapy was shown to be associated with reduction in serum TNC level, suggesting that TNC may reflect ongoing myocardial damage (121).

Since TNC is induced by various inflammatory mediators, it may also reflect the disease activities of inflammatory cardiovascular diseases including Kawasaki disease (122, 123) and cardiac sarcoidosis (124). TNC is expressed locally in the tissue of coronary atherosclerosis (125) and abdominal aortic aneurysm (112, 126), and its expression is elevated in serum of patients with these diseases (127). The serum level of TNC is elevated in patients with acute aortic dissection and its elevated level is associated with acute mortality (128), as well as chronic prognosis (129, 130). TNC is also elevated in cerebrospinal fluid after subarachnoidal hemorrhage and may predict the development of cerebral vasospasm (131). Elevated serum TNC is not only associated with specific diseases but also with the mortality and the development of cardiovascular diseases in patients with chronic kidney disease (132) and it is also associated with major adverse cardiovascular events and death in individuals with type 2 diabetes mellitus (133). In addition to the serum level, local deposition of TNC may serve as a marker of tissue damage, as demonstrated in animal models of myocarditis (134) and myocardial infarction (135). Therefore, quantitative detection of systemic and local levels of TNC may have a clinical value for monitoring inflammation and tissue damage both in acute and chronic diseases in order to realize precision medicine for better outcomes by optimizing the clinical practice for individual requirement.

Considering the fact that the structure of TNC can be altered in a disease-specific manner, domain-specific detection of TNC may also have a clinical value (92). For example, isoform-specific expression of TNC was demonstrated in the lung tissue of experimental pulmonary hypertension (136) and in the serum of the patients (137). This means that care should be taken which isoform of TNC is being measured to evaluate its significance as a biomarker in a particular clinical setting, as well as the normal range of TNC concentration. Because of the significance of the different TNC isoforms, domain-specific monoclonal antibodies for TNC would have potential clinical values both as diagnostic tools to evaluate the disease conditions, and as therapeutic tools to target a particular function of TNC or a particular tissue that expresses the corresponding TNC isoform (92).

## Conclusions

TNX and TNC have distinct roles in physiological and pathological conditions. In a physiological condition, TNX is involved in the structural integrity of collagen fibrils. TNX also has a tumor suppressor role, a proangiogenic property, a role in osteoclast differentiation, and a role in TGF-β activation. On the other hand, in a pathological condition such as TNX deficiency, its absence causes clEDS with major clinical features such as hyperextensible skin without atrophic scarring, generalized joint hypermobility and easy bruising. Interestingly, TNX deficiency is involved in pain and fibrosis. The underlying molecular mechanisms for pain and suppression of fibrosis caused by TNX deficiency need to be elucidated in more detail.

The physiological role of TNC is yet to be clarified. Although genetic deletion of TNC in mice resulted in no gross abnormality of the animals, the possibility remains that TNC plays a role in cell differentiation and tissue organization during embryogenesis. On the other hand, accumulating evidence indicates that TNC is re-expressed and actively participates in the pathogenesis of various diseases with tissue damage. The context-dependent function of TNC, possibly due to its modular structure and multiple binding partners, makes it difficult to interpret the experimental results as to whether expression of TNC is detrimental or beneficial. Nonetheless, expression of TNC seems to be a sensitive marker for tissue damage both in cardiovascular and non-cardiovascular diseases including cancer. Considering the wide range of physiological and pathophysiological functions of tenascins and their specific expression patterns, basic and clinical studies of tenascin family would be fruitful for delineating their precise roles and their clinical implications both in normal and abnormal conditions.

## Author Contributions

KM and HA designed and wrote this manuscript. All authors contributed to the article and approved the submitted version.

## Funding

This work was supported by the Japan Society for the Promotion of Science KAKENHI grant number 19K08470 to KM and 19H03743 to HA from the Ministry of Education, Culture, Sports, Science and Technology of Japan.

## Conflict of Interest

The authors declare that the research was conducted in the absence of any commercial or financial relationships that could be constructed as a potential conflict of interest.

## Abbreviations

BDNF, brain-derived neurotrophic factor; BNP, B-type natriuretic peptide; circRNA, circular RNA; clEDS, classical-like Ehlers-Danlos syndrome; CNS, central nervous system; DRG, dorsal root ganglion; ECM, extracellular matrix; EDS, Ehlers-Danlos syndrome; EGF, epidermal growth factor; EMT, epithelial-mesenchymal transition; FBG, fibrinogen; FGFs, fibroblast growth factors; FNIII, fibronectin type III; HFCD, high levels of phosphorus and calcium; HFpEF, heart failure with preserved ejection fraction; lncRNA, long non-coding RNA; MMPs, matrix metalloproteinases; PDGFs, platelet-derived growth factors; PNS, peripheral nervous system; SLE, systemic lupus erythematosus; SNPs, single nucleotide polymorphisms; sTNX, serum form of TNX; TGF-β, transforming growth factor-β; TLR4, Toll-like receptor 4; TNC, tenascin-C; TNR, tenascin-R; TNW, tenascin-W; TNX, tenascin-X; *Tnxb*, mouse tenascin-X gene; *TNXB*, human tenascin-X gene; TNY, tenascin-Y; VEGF-B, vascular endothelial growth factor, B; VEGFR-1, vascular endothelial growth factor receptor 1; VUR, vesicoureteral reflux.
